# The National Cancer Institute of Canada Clinical Trials Group MAP.3 trial: an international breast cancer prevention trial

**DOI:** 10.3747/co.2007.117

**Published:** 2007-06

**Authors:** H. Richardson, D. Johnston, J. Pater, P. Goss

**Affiliations:** * National Cancer Institute of Canada Clinical Trials Group, Queen’s University, Kingston, Ontario; † Harvard Medical School, Massachusetts General Hospital Cancer Center, Boston, Massachusetts, U.S.A

**Keywords:** Aromatase inhibitors, breast cancer risk reduction, chemoprevention

## Abstract

Several large phase iii trials have demonstrated that tamoxifen—and more recently, raloxifene—can effectively reduce the incidence of invasive breast cancer by 50%. However, these selective estrogen receptor modulators can also be associated with several rare, but serious, adverse events. Recently, the third-generation aromatase inhibitors (ais) have demonstrated excellent efficacy in adjuvant breast cancer trials, and they show particular promise in the breast cancer prevention setting. The National Cancer Institute of Canada Clinical Trials Group (ncic ctg) has developed a randomized phase iii study to determine the efficacy of an ai (exemestane) to reduce the incidence of invasive breast cancer in postmenopausal women at an increased risk for developing breast cancer. The ncic ctg map.3 (ExCel) trial is a double-blind placebo-controlled multicentre, multinational trial. Based on the known preclinical and clinical profile of the ais, a greater reduction in breast cancer incidence with fewer side effects is hypothesized with this class of agents than with tamoxifen or raloxifene.

## 1. INTRODUCTION

Breast cancer is one of the most frequently diagnosed malignancies in women worldwide. In North America, it represents about 30% of all cancers and about 20% of all cancer-related deaths. In the United States and Canada respectively, more than 200,000 and 20,000 new breast cancer cases are diagnosed annually, and beginning in the 1980s and continuing until very recently, the incidence of breast cancer was rising at a rate of approximately 2% per year^1^,^2^.

The epidemiology of breast cancer is strongly influenced by genetic and environmental factors. Compared with other women, women with breast cancer are about twice as likely to have a first-degree relative with breast cancer, suggesting that the genetic factors are important determinants of disease risk^3^–^5^. However, large international differences in the rates of breast cancer and changes in the rates of disease in migrants from low-risk to high-risk countries suggest that environmental factors also play an important causative role^6^–^8^.

Decades of epidemiologic and laboratory research have also identified that hormonal exposures are important in the complex origins of breast cancer. Hormonal events such as early age at menarche, late age at parity, and late onset of menopause can all increase breast cancer risk^9.10^. To date, most research has focused on endogenous sex steroid exposure, and higher levels of estrogen and androgens have been shown to be associated with a modest increase in the risk of breast cancer in pre- and postmenopausal women^11^–^13^. Interest in studying the roles of other hormones such as prolactin^14^ and the insulin–insulin-like growth factor (igf) axis^15^ is increasing, based on recent preclinical studies demonstrating the important role that igf-1 plays in stimulating the growth of prostate and breast cancer cells alike^16^.

Many of the established risk factors currently identified for breast cancer cannot be easily modified. However, improved diet and reduced body weight, alcohol intake, hormone use, and mammographic density are examples of factors that can^17^–^21^. At the 29th annual San Antonio Breast Cancer Symposium, Dr. Ravdin and colleagues from the National Cancer Institute and Harbor University of California, Los Angeles Medical Center, reported on an overall 7% decline in breast cancer incidence from 2002 to 2003^2^, based on data from the Surveillance Epidemiology and Endpoints Results database. He noted that the decline was greatest in women over 50 years of age. Similar data have also been observed in California over the same time period and for 2004^22^.

Dr. Ravdin and his team speculate that the dramatic reduction in use of hormone replacement therapy (hrt) in 2002 may be the best explanation for the reduction in breast cancer occurrence. Following the disappointing findings in 2002 from the Women’s Health Initiative Study of combination estrogen and progestin, which demonstrated a statistically significant increase (24%) in the breast cancer risk in the treatment arm, hrt prescribing patterns were significantly altered^20^. Clarke and colleagues showed that hormone therapy use dropped 68% between 2001 and 2003 in California, and shortly thereafter breast cancer rates dropped by 10%–11%^22^. That decline was sustained in 2004.

Breast cancer may take decades to grow, but many of these cancers are believed to be fuelled by hormones such as estrogen. By cutting the fuel supply, tumour growth is slowed down substantially—and possibly stopped. In turn, these tumours may be harder to detect by mammography. If estrogen can act as a promoter of tumour growth, it is possible that a change in hrt use could translate into a full 7% reduction in breast cancer occurrence 1 year later, based on the high prevalence of hrt use in postmenopausal women before 2002^2^.

## 2. BREAST CANCER CHEMOPREVENTION

The foregoing data and other epidemiologic studies have prompted researchers to hypothesize about strategies that may reduce the frequency of breast cancer. One logical strategy is to investigate agents capable of interfering with the initiation or promotion of the disease—in other words, breast cancer chemoprevention.

To date, chemoprevention research has focused on strategies directed at antagonizing the effects of estrogens, because these hormones are known to play a key role both in the development of the normal breast and in the pathogenesis of breast cancer^23^,^24^. In principle, at least two pharmacologic approaches may be used to antagonize the effects of estrogen in the breast. The first is to inhibit estrogen binding to its receptor by using selective estrogen receptor modulators (serms—tamoxifen and raloxifene). An alternative strategy of antagonizing (reducing) the effects of estrogen is to inhibit estrogen synthesis with an aromatase (estrogen synthetase) inhibitor (ai).

### 2.1 Inhibition of Estrogen Binding

Tamoxifen and raloxifene have both been shown to reduce the incidence of invasive breast cancer by up to 50% in pre- and postmenopausal women at high risk^25^–^27^. Both drugs only reduce the incidence of estrogen receptor–positive breast cancer, consistent with their mode of action as serms. As a result, tamoxifen was approved by the U.S. Food and Drug Administration (fda) for short-term reduction in the incidence of ductal carcinoma *in situ* (dcis) and invasive breast cancer in women at increased risk. Raloxifene is currently under review by the fda for approval as a second chemopreventive agent for women at high risk of developing invasive breast cancer. In fact, tamoxifen and raloxifene both appear effective in reducing breast cancer risk in “all risk” individuals as well, but approval for tamoxifen was granted by the fda only for use in high-risk women because of its complicated therapeutic index. In particular, tamoxifen can cause rare, but serious, adverse events, including endometrial cancer and thromboembolic disease, especially in older postmenopausal women^28^.

### 2.2 Inhibition of Estrogen Synthesis

The effect of ais on risk of breast cancer in postmenopausal women is currently under study. The National Cancer Institute of Canada Clinical Trials Group (ncic ctg) map.3 trial is one such study designed to examine the efficacy of exemestane versus placebo in postmenopausal women at increased risk of developing breast cancer. The International Breast Cancer Intervention Study 2 (ibis 2), initiated in 2004, is the only other large, phase iii trial designed to evaluate the efficacy of anastrozole as compared with placebo in preventing invasive breast cancer. It is underway in a similarly high-risk population in the United Kingdom^29^.

## 3. AROMATASE INHIBITORS AS POTENTIAL CHEMOPREVENTIVE AGENTS

Targeting and reducing estrogen synthesis is a way of preventing estradiol from stimulating the estrogen receptor and of reducing the formation of cancer-causing catechol metabolites of estrogen. To that end, ais were developed.

Aromatase is the enzyme complex responsible for the final step in estrogen biosynthesis: the conversion of androgens to estrogens. The third-generation ais letrozole, anastrozole, and exemestane are all approved for use in postmenopausal women with estrogen receptor–positive metastatic breast cancer that has progressed after tamoxifen or failed to respond to tamoxifen^30^–^33^, or as initial therapy in treatment-naïve women with receptor-positive metastatic disease. In addition, the fda and Health Canada have approved anastrozole, exemestane, and letrozole for use as adjuvant therapy for postmenopausal women with hormone receptor–positive breast cancer following varying periods of treatment with tamoxifen.

At least eight adjuvant trials are currently testing ais in early-stage postmenopausal receptor-positive breast cancer. Published data from four large phase iii double-blind randomized adjuvant trials comparing third-generation ais with tamoxifen or placebo after 5 or fewer years of tamoxifen are currently available.

In the Arimidex, Tamoxifen Alone or in Combination (atac) trial, 9366 patients were randomly assigned to receive anastrozole and placebo, tamoxifen and placebo, or anastrozole and tamoxifen combined. Disease-free survival was significantly lengthened when the anastrozole group was compared with the tamoxifen group (absolute risk reduction: 2.7%; *p* = 0.013) after a median follow-up of 47 months. Importantly, the incidence of new contralateral primary breast cancer was significantly lower in the anastrozole group than in the tamoxifen group [odds ratio (or): 0.42; *p* = 0.007]^34^.

The Intergroup Exemestane Study (ies) randomly assigned 4742 women who had received 2–3 years of tamoxifen to continue tamoxifen for a total of 5 years or to switch to exemestane to complete a 5-year course of hormonal therapy. After a median follow-up of 56 months, a significant improvement in disease-free survival was observed in the exemestane group [hazard ratio (hr): 0.76; 95% confidence interval (ci): 0.66 to 0.88], together with a significant reduction in contralateral breast cancer events (hr: 0.56; 95% ci: 0.32 to 0.97) 35 and a modest improvement in overall survival 36.

The ncic ctg ma.17 trial involved 5187 postmenopausal women who had taken tamoxifen for 5 years and who were disease free at time of study entry. They were randomly assigned to receive 5 years of letrozole or 5 years of placebo. The study was halted by the Data Safety Monitoring Committee after a median of 2.4 years because of a significant reduction in breast cancer events in the treatment arm^37^. More recently, the study demonstrated an overall benefit in distant disease-free survival and a survival advantage in the subset of women on the trial who had node-positive disease. The incidence of contralateral cancers was also lower in the letrozole group, although the difference was not statistically significant^38^.

The Breast Cancer International Study Group involved approximately 8000 women and had four treatment arms: tamoxifen alone for 5 years, letrozole alone for 5 years, tamoxifen for 3 years followed by letrozole for 2 years, and letrozole for 3 years followed by tamoxifen for 2 years. A recent analysis comparing letrozole to tamoxifen treatment showed that the reduction in risk of recurrence or death was 19% lower in the letrozole-alone group after a median of 28 months of follow-up. Comparisons of the switched arms are not yet available^39^.

Based on results from these large trials and other smaller randomized trials, the Technology Assessment of Aromatase Inhibitors Status Report 2004 from the American Society of Clinical Oncology (asco) recommends that, to lower the risk of recurrence, adjuvant therapy for postmenopausal women with hormone receptor–positive breast cancer should include ais. The optimal timing and duration of ai therapy has yet to be established^40^.

The thus-far convincing evidence that the ais are superior to tamoxifen in the treatment of breast cancer has suggested that they will also perhaps prove to be superior in the chemopreventive setting.

Exemestane is a third-generation irreversible steroidal aromatase inactivator, structurally related to the natural substrate androstenedione. In postmenopausal women, exemestane is capable of inhibiting aromatase action by more than 95%. The reduction in contralateral breast cancer incidence in the atac and ies trials is especially promising in this regard.

In addition to the clinical studies, a significant body of data on the chemopreventive properties of ais comes from preclinical studies^41^–^44^.

## 4. NCIC CTG MAP.3

The map.3 (ExCel) trial (www.excelstudy.com) is a randomized double-blind placebo-controlled, multicentre, multinational trial sponsored by the ncic ctg and supported by Pfizer Inc. Based on the known pre-clinical and clinical profile of the ais, a greater reduction in breast cancer incidence is hypothesized with this class of agents than with tamoxifen or raloxifene.

### 4.1 Study Design

The initial intent was to compare exemestane (25 mg) in combination with a cyclooxygenase enzyme 2 (cox-2) inhibitor, celecoxib (400 mg); exemestane (25 mg) in combination with a placebo; and a placebo (2 tablets) in a phase iii randomized trial, in 5100 post-menopausal women at increased risk of developing breast cancer. The rationale for incorporating celecoxib into the trial design was based on observations that cox-2 is overexpressed in breast cancer and in pre-invasive breast lesions^45^–^47^, and that a meta-analysis of 14 cohort and case-control studies had found a combined relative risk of 0.82 (95% ci: 0.75 to 0.89) for developing breast cancer in women who had a history of taking cox-2 inhibitors^48^. Further-more, in preclinical studies, a synergistic effect appeared to exist between celecoxib and exemestane, resulting in a greater ability to prevent new tumours and to reduce the existing tumour burden in animals when the drugs were combined^49^. At the time that the map.3 protocol was developed, celecoxib was considered to be well tolerated and safe.

Each of the three arms was to be tested separately and consecutively. Celecoxib–placebo was to be prescribed for a total of 3 years, and exemestane–placebo was to be prescribed for a total of 5 years. The original map.3 protocol was activated in 2004, and the first 35 participants were enrolled between September and November 2004.

Unfortunately, in September 2004, early results from a colorectal adenoma chemoprevention trial (approve)^50^ showed an elevated cardiovascular risk in subjects with a history of colorectal adenomas who were taking rofecoxib (Vioxx: Merck, Whitehouse Station, NJ, U.S.A.), which, until that point, had been considered a very promising chemopreventive agent for colorectal adenomas. In late December, data from the Adenoma Prevention with Celecoxib trial^51^ showed a similar association between celecoxib and cardiovascular risk. Consequently, the map.3 steering committee decided to halt the trial and revise the protocol.

The revised map.3 study now compares exemestane to placebo in a 1:1 ratio in 4560 postmenopausal women who are 35 years of age or older and at increased risk for the development of breast cancer. For the purposes of this protocol, “increased risk” is defined as being over the age of 60, or having a Gail score greater than 1.65, or having a prior atypical breast biopsy (atypical ductal hyperplasia, atypical lobular hyperplasia, or lobular carcinoma *in situ*), or having a prior diagnosis of dcis that was treated with a mastectomy. Women are stratified on their Gail score (≤ 2 vs. >2) and current low-dose (<100 mg daily) aspirin use (yes vs. no) before being randomly allocated to the treatment or placebo group.

### 4.2 Rationale for Placebo

Despite the fact that tamoxifen has been approved as a means to reduce breast cancer risk in women who would be eligible for this trial, it was decided that the use of a placebo control arm was justified for several reasons:

- Although tamoxifen is approved for the indication of reducing the short-term incidence of breast cancer, many women whose level of risk would qualify them for the prescription of tamoxifen refuse the drug because of its toxicity profile^52^–^55^. Raloxifene is another option that women may consider for breast cancer risk reduction, although it is not yet approved for that indication. However, although the risk profile for raloxifene may be better than that for tamoxifen, it is still associated with increased risk of thromboembolic events and decreased sexual function^27^. Therefore, there remains a population of women eligible for this trial who have chosen or will choose, even after appropriate counselling, to avoid taking tamoxifen or raloxifene. These women may well wish to enter a placebo-controlled trial where the agents under study may have more favourable toxicity profiles.
- The asco Technology Assessment of Pharmacologic Interventions for Breast Cancer Risk Reduction Including Tamoxifen, Raloxifene and Aromatase Inhibition^56^ concluded that “placebo controls are appropriate for breast cancer risk reduction trials since no intervention has been demonstrated to favorably impact net health or survival.” Although the map.3 trial is not expected to demonstrate an impact on survival, the results may well indicate a more favourable therapeutic ratio for exemestane than for tamoxifen or raloxifene.
- The placebo arm will allow for a true determination of efficacy in reducing invasive breast cancer, of adverse effects, and of impact on overall and menopausal-specific quality of life.

### 4.3 Study Procedures

Women enrolled in the map.3 research study will have a bone-mineral density test and a mammogram before being randomized. Women can be enrolled at centres in Canada, the United States, or Spain. Participants will be asked to return to their local study centre twice during the first year, at 6 and 12 months, and then annually for follow-up visits for the remaining 4 years of the study. At each visit, participants will be given a new supply of the study medications and will be asked to answer questions about their quality of life and about any illnesses or discomfort they may have experienced since their last visit. At each annual visit, participants will undergo a physical and health exam and a mammogram. At three different times during the study, serum samples will also be taken for hormone testing. If additional consent has been given for dna testing, a blood sample taken at baseline will be stored for future genetic testing.

### 4.4 Study Goals and Population

The main objective of the map.3 trial is to compare the incidence of breast cancer in the two treatment groups. Information will also be recorded and compared between treatment groups on clinical bone fractures, cardiovascular events, quality of life, tolerability and safety, and incidences of other malignancies. A companion study to evaluate the long-term effects of exemestane on bone density and bone biomarkers is planned for a subset of participating sites.

Some of the biggest challenges of conducting a breast cancer prevention trial include recruiting women from the general population and defining the groups at high-risk for the development of breast cancer that are most eligible for chemoprevention. Well established cancer cooperative groups with affiliated clinical research centers in North America, Europe, and Australia are available to help with recruitment of cancer patients into therapeutic trials. However, that model is not so easy to replicate for cancer prevention trials. The clinicians that typically see well women are more likely to be primary care physicians or other internal specialists such as gynecologists. However, primary care physicians in particular do not appear to be comfortable prescribing chemoprevention medication^57^–^59^, and based on results from a recent national survey in the United States, the decision to prescribe tamoxifen was greatly affected by logistics and the ability of the physician to determine eligibility^59^.

For the map.3 trial to be feasible, the ncic ctg had to assemble a consortium of clinical researchers in Canada and the United States who were committed to cancer prevention research and who had participated in earlier prevention trials with the Women’s Heath Initiative (whi) or the National Surgical Adjuvant Breast and Bowel Project. Nonetheless, recruiting to chemoprevention trials is still very challenging, even for experienced research centres. To be successful, each site must consider numerous recruitment strategies. Some of the most successful strategies to date on map.3 include mass mailings, targeting of high-risk screening clinics, and targeting of “enriched” lists (that is, women who participated in the whi observational study and expressed an interest in participating in future cancer prevention research).

Cancer prevention trials tend to be dauntingly large, particularly if cancer is the primary endpoint. The large sample size is necessary because cancer is a very rare occurrence in the general population, and there is a need to complete the study in a reasonable period of time (that is, 5 years). Selecting a study sample that has a higher-than-average risk of developing cancer is one strategy to increase the event rate in a fixed period and thereby reduce the required sample size.

Risk prediction models can be included in the design of chemoprevention studies to help identify high-risk populations for an assessment of the effects of interventions and construction of risk–benefit indices for preventive interventions^60^. The Gail model is one such existing model that has modest predictive ability^61^. Based on six well-established risk factors (age, age at menarche, age at first birth, first-degree family history of breast cancer, prior breast biopsy, and race), the Gail model calculates a woman’s 5-year risk (probability) of developing breast cancer and compares it with the average risk for a woman of the same age and race or ethnicity from the general U.S. population^62^.

Age is one of the most important risk factors for breast cancer. An average 60-year-old woman has a Gail score of 1.8%; an average 35-year-old woman has a Gail score of 0.3%. By designing a prevention trial with a minimum Gail score requirement of 1.66% for women who are under 60 years of age and have no history of benign breast disease, the expected annual incidence rate (event rate) in the placebo group (0.60%) will be more than 6 times the annual incidence rate in the general population (0.10%)^63^. Admittedly, estimates of risk could be greatly improved with the addition of specific genetic profiles with validated candidate genes, of lifestyle risk factors, and of mammographic density^64^. However, the appeal of the Gail model is that it is reliable, cost-efficient, and easy to use in large-scale breast cancer prevention trials, in which thousands of women need to be screened to determine eligibility.

## 5. SUMMARY

In the 15 years since the first breast cancer prevention trial was initiated^25^, several large phase iii trials have consistently demonstrated that tamoxifen—and more recently, raloxifene—can effectively reduce the incidence of invasive breast cancer by 50%. However, these serms can also be associated with several rare, but serious adverse events, which may explain their low uptake for chemopreventive purposes.

Currently, a great deal of interest exists in the third-generation ais. These agents have demonstrated excellent efficacy in adjuvant breast cancer trials and show particular promise in the breast cancer prevention setting, based on the significantly higher reduction rates for contralateral breast cancer in the groups of women on an ai than in those on tamoxifen^34^,^35^.

The ais are generally well tolerated. Side effects are similar to those of decreased estrogen—hot flashes, increased blood pressure, and thinning of bones, for example. However, the toxicity profile of exemestane may have advantages over the serms and the other ais. To date, no evidence has been uncovered that, as compared with placebo or tamoxifen, exemestane is associated with any significant increase in cardiovascular disease or adverse effect on lipid profile^65^. In addition, because of the weak androgenic and anabolic properties of the principal metabolite of exemestane, 17-hydroexemestane^66^, exemestane may have fewer negative effects on bone metabolism than do other ais.

Although it is too early to speculate about the overall efficacy of ais in chemoprevention and about their overall safety profile, map.3 participants can be assured that their safety will be closely monitored and regularly reviewed by a Data Safety and Monitoring Committee. One important question that will arise from the current series of adjuvant and chemo-prevention trials of ais is whether distinct pharmacodynamic effects are associated with the nonsteroidal ais (anastrozole and letrozole) and the steroidal aromatase inactivator (exemestane) that would favour one type of ai over another in the prevention setting.
